# A Rare Case of Neuromyelitis Optica Spectrum Disorders With Unknown Fever and Subacute Cognitive Decline With Normal Images

**DOI:** 10.7759/cureus.24950

**Published:** 2022-05-12

**Authors:** Kento Furuya, Naoya Itoh

**Affiliations:** 1 Department of Emergency, Shizuoka General Hospital, Shizuoka, JPN; 2 Division of Infectious Diseases, Aichi Cancer Center Hospital, Nagoya, JPN

**Keywords:** hashimoto’s encephalopathy, altered consciousness, magnetic resonance imaging, fever of unknown origin, anti-aquaporin 4 antibody, neuromyelitis optica spectrum disorder

## Abstract

We report the case of a 69-year-old Japanese man who came to our hospital with a chief complaint of fever and cognitive decline for three weeks. There were no neurological abnormalities other than the decreased level of consciousness. He developed urinary retention after admission, so we performed a lumbar puncture, although his head and neck magnetic resonance imaging (MRI) showed no abnormal findings. The cerebrospinal fluid (CSF) examination showed albuminocytologic dissociation and the anti-aquaporin 4 antibody was positive. Thus, we diagnosed him with neuromyelitis optica spectrum disorder (NMOSD). NMOSD is an autoimmune disease that causes demyelination. The clue to diagnosing NMOSD is demyelinating findings on MRI. Therefore, it is difficult to diagnose NMOSD if there are no abnormalities on the images. However, abnormal MRI findings are not necessary for the diagnosis of NMOSD. Thus, NMOSD cannot be ruled out even if MRI findings are normal and the real clue to diagnosing NMOSD is the anti-aquaporin 4 antibody.

## Introduction

Neuromyelitis optica spectrum disorder (NMOSD) is a demyelinating autoimmune disease characterized by optic neuritis and transverse myelitis [1]. Abnormal magnetic resonance imaging (MRI) triggers suspicion of NMOSD, and up to 85% of NMOSDs have abnormal MRI of the head or spinal cord [2]. We report a case of atypical NMOSD with fever and subacute cognitive symptoms and no abnormalities on MRI. Although MRI findings are generally the clue to the diagnosis of NMOSD [2,3], abnormal imaging findings of the head or spine are not necessary for the diagnostic criteria of NMOSD [1]. And autoimmune diseases, such as systemic lupus erythematosus (SLE) and Hashimoto's encephalopathy, can cause fever and loss of consciousness [2,4] but are difficult to distinguish from NMOSD based on symptoms alone [5,6]. We should not rule out NMOSD even if the MRI findings are normal and the key to the diagnosis of NMOSD was the anti-aquaporin 4 (anti-AQP4) antibody.

## Case presentation

A 69-year-old Japanese man presented with a three-week history of continuous fever and acute cognitive decline. Acetaminophen and oral antibiotics were prescribed, but the fever persisted. Simultaneously, the patient’s family reported that "his driving suddenly became rough" and "he suddenly became forgetful." 

At the time of admission, the patient’s level of consciousness was mildly diminished (Glasgow Coma Scale E4V4M6) and his temperature was 38.3 °C. The general examination findings were unremarkable. Moreover, a neurological examination indicated normal cerebral nerve function, including normal visual acuity, and unremarkable results on visual field tests. Deep tendon reflexes, motor, and sensory were also unaffected. He scored 16 points on the Mini-Mental State Examination (MMSE). His blood test results, including those of thyroid function tests, were almost normal (Table 1).

**Table 1:** Laboratory findings on the day of admission.

Parameter	Lab value
White blood cell (/µL)	5,500
Red blood cell (/µL)	410
Hemoglobin (/dL)	13.8
Platelet (/µL)	14.8 x 10^4^
Blood urea nitrogen (mg/dL)	11
Creatinine (mg/dL)	0.6
Albumin (g/dL)	3.7
Total bilirubin (g/dL)	0.9
Alanine transaminase (U/L)	17
Aspartate aminotransferase (U/L)	12
Lactate dehydrogenase (U/L)	151
C-reactive protein (mg/dL)	0.02
Free T4 (pg/dL)	1.23
Blood sugar (mg/dL)	244
Hemoglobin A1c (%)	8.8

**Figure 1:**
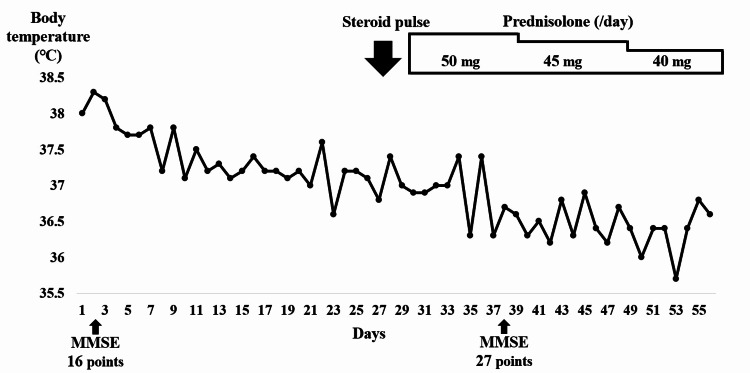
Hospitalization progress chart depicting the patient’s clinical course.

He was diagnosed with diabetes eight years ago, and his diabetes control was poor (HbA1c, 8.8%). Computed tomography of the head, neck, chest, and abdomen was unremarkable. After admission, the patient continued to have a fever of 37‐38 °C (Figure 1), but we did not use antibiotics. On day 1 of hospitalization, blood, sputum, and urine cultures were negative. On day 4, lumbar puncture showed 141 mg/dL protein in the cerebrospinal fluid (CSF) and a lymphocyte count of 4/mm^3^. The CSF adenosine deaminase level was 7.4 U/L, and the CSF culture was negative.

**Figure 2:**
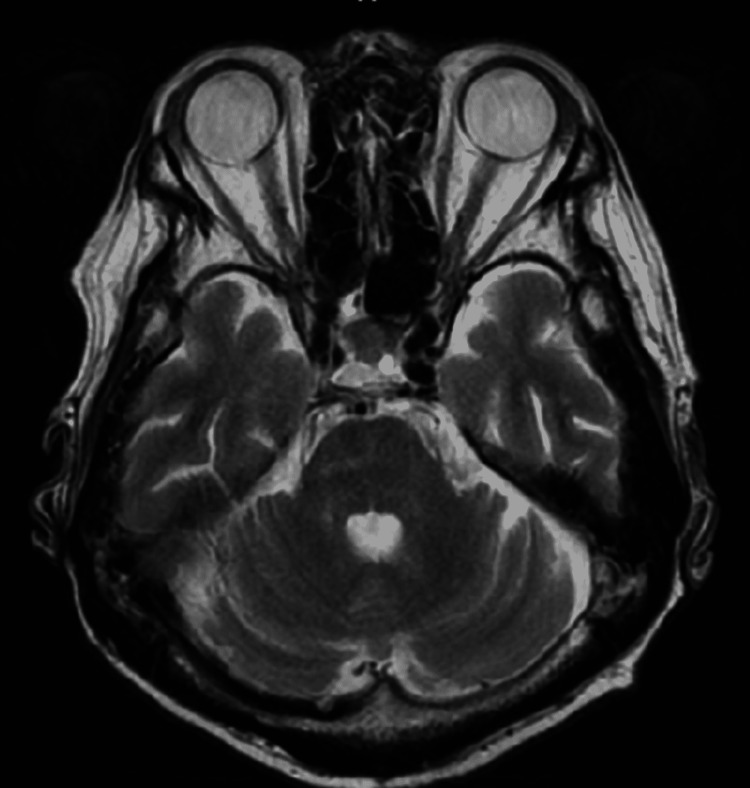
T2-weighted MRI of the head shows no abnormalities, including the optic nerve.

Encephalitis, central nervous system infection, syphilis, malignant lymphoma, vitamin deficiency, Hashimoto’s encephalopathy, vasculitis, and autoimmune diseases were considered possible causes of his illness; thus, a head MRI was performed, although no abnormal findings were found (Figure 2).

The anti-thyroglobulin antibody and anti-thyroid peroxidase antibody were positive, but serum testing excluded syphilis, Sjogren’s syndrome, SLE, vasculitis, and paraneoplastic neurologic syndrome (Table 2). On day 10, dysuria developed, and an indwelling urethral balloon was inserted on day 16. Rectal examination revealed decreased contraction of the anal sphincter.

**Table 2:** Laboratory findings after admission. PR3-ANCA: proteinase-3-antineutrophil cytoplasmic antibodies, MPO-ANCA: myeloperoxidase-antineutrophil cytoplasmic antibodies, SSA: Sjogren's syndrome.

Parameter	Lab value
Anti-thyroglobulin antibody (IU/mL)	65.3
Anti-thyroid peroxidase antibody (IU/mL)	95.4
Vitamin B1 (ng/mL)	86.4
Vitamin B12 (pg/mL)	11,600
Anti-nuclear antibody	1:40
Anti-Ro/SSA antibody (U/mL)	<10.0
Anti-La/SSA antibody	<10.0
PR-3 ANCA (U/mL)	<1.0
MPO-ANCA (U/mL)	<1.0
Soluble interleukin-2 receptor (U/mL)	151
Rapid plasma reagin (R.U.)	0.5
Anti-treponema pallidum (U/mL)	1.6

We suspected a vesico-rectal disorder and considered an MRI of the lumbar region but were unable to perform it due to a history of posterior fusion surgery for a lumbar hernia. At that time, stuttering began. On day 14, the spinal fluid was reexamined; a protein level of 194 mg/dL, a lymphocyte count of 4/mm^3^, and protein-cell dissociation were still observed, and the spinal fluid oligoclonal band was positive.

**Figure 3:**
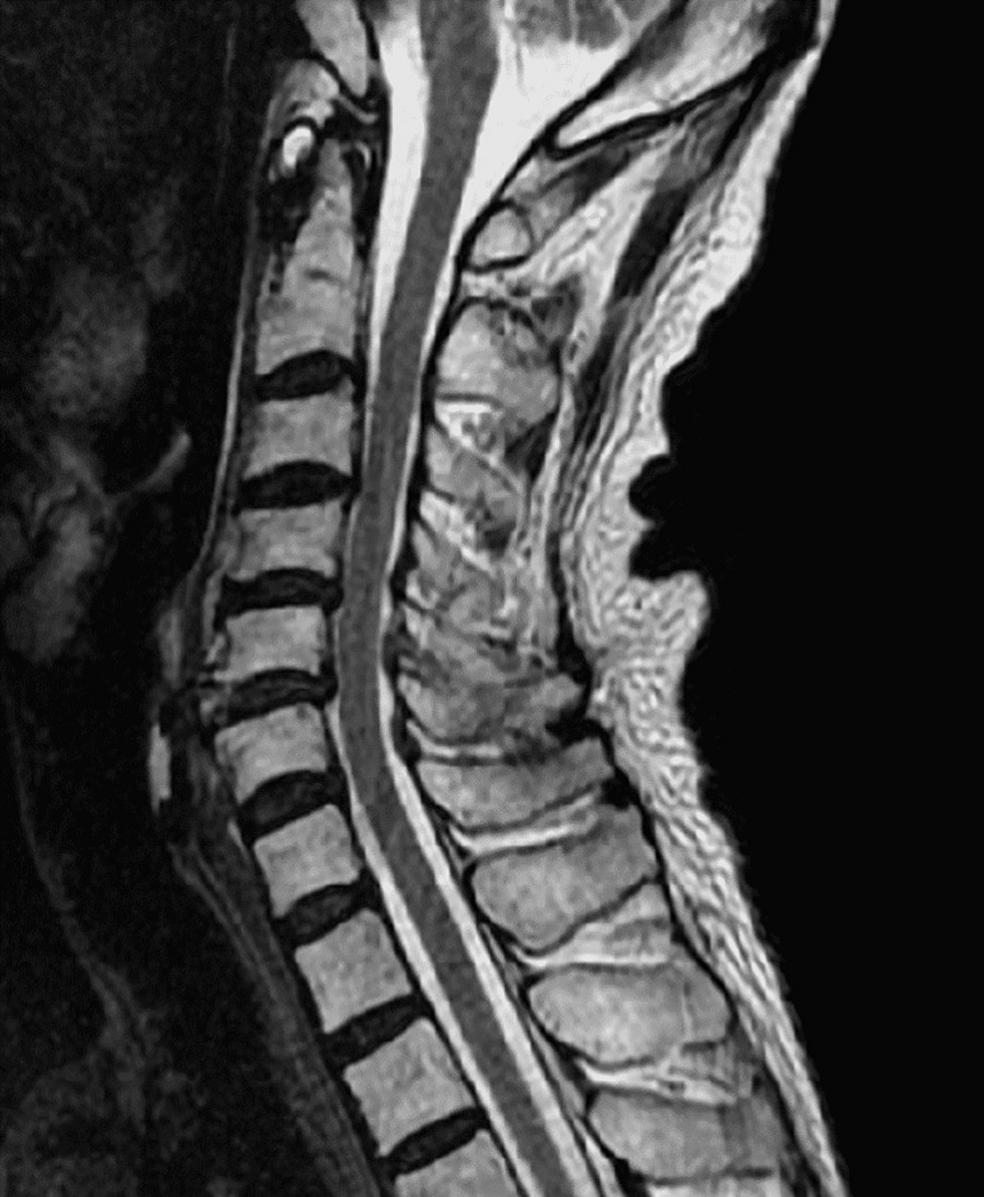
T2-weighted MRI of the neck does not show demyelination.

Consequently, multiple sclerosis, neuromyelitis optica (NMO), or Hashimoto’s encephalopathy were suspected. We tested for anti-AQP-4 antibody, which was positive. MRI of the neck and head indicated cervical herniation but no evidence of demyelination (Figure 3). Thus, NMOSD was diagnosed.

Steroid pulse therapy (methylprednisolone, 1,000 mg/day) was started on days 30-32, and prednisolone 50 mg/day (1 mg/kg/day) was started on day 33. After the initiation of steroid therapy, the patient no longer had a fever (Figure 1). On day 38, his MMSE score was 27 points, indicating cognitive function recovery. On day 43, the urethral balloon was removed, and the voiding function recovered. The prednisolone dosage was reduced to 45 mg/day on day 44 and 40 mg/day on day 51, but there was no relapse of symptoms. The patient was discharged on the 56th day.

## Discussion

NMOSD is a demyelinating autoimmune disease characterized by optic neuritis and transverse myelitis [1]. Although MRI findings are generally the clue for the diagnosis of NMOSD [2], we report a case of atypical NMOSD with fever, subacute cognitive symptoms, and no MRI abnormalities. The key to diagnosis, in this case, was the presence of the anti-AQP4 antibody.

Imaging studies, particularly MRI, play an important role in the diagnosis of NMO/NMOSD. Optic neuritis, myelitis, and encephalopathy are symptoms of NMO and NMOSD; up to 85% of patients with NMO or NMOSD have abnormalities on head MRI, and 72.2% have abnormalities on spinal MRI [2]. In addition, spinal cord lesions are characterized by a "long cord lesion" that extends over three or more vertebrae and are more common in the thoracic spine than in the lumbar spine [1,3]. And one study reported that the sensitivity of MRI is 82% and the specificity is 91% in NMOSD cases [2]. Thus, imaging tests can provide major clues for the diagnosis of NMOSD. However, because positive imaging findings are not required for the diagnostic criteria (Table 3) [1], the NMOSD diagnosis must be considered even in the absence of abnormal MRI findings, as in the present case.

**Table 3:** NMOSD diagnostic criteria for adult patients. NMOSD: neuromyelitis optica spectrum disorder, AQP-4: aquaporin 4, LETM: longitudinally extensive transverse myelitis [1].

Diagnostic criteria for NMOSD with AQP4-IgG
1. At least one core clinical characteristic
2. Positive test for AQP4-IgG using best available detection method (cell-based assay strongly recommended)
3. Exclusion of alternative diagnoses
Diagnostic criteria for NMOSD without AQP4-IgG or NMOSD with unknown AQP4-IgG status
1. At least two core clinical characteristics occurring as a result of one or more clinical attacks and meeting all of the following requirements
a. At least 1 core clinical characteristic must be optic neuritis, acute myelitis with LETM, or area postrema syndrome
b. Dissemination in space (two or more different core clinical characteristics)
c. Fulfillment of additional MRI requirements, as applicable
2. Negative tests for AQP4-IgG using the best available detection method, or testing unavailable
3. Exclusion of alternative diagnoses
Core clinical characteristics
1. Optic neuritis
2. Acute myelitis
3. Area postrema syndrome: an episode of otherwise unexplained hiccups or nausea and vomiting
4. Acute brainstem syndrome
5. Symptomatic narcolepsy or acute diencephalic clinical syndrome with NMOSD-typical diencephalic MRI lesions
6. Symptomatic cerebral syndrome with NMOSD-typical brain lesions
Additional MRI requirements for NMOSD without AQP4-IgG and NMOSD with unknown AQP4-IgG status
1. Acute optic neuritis: requires brain MRI showing (a) normal findings or only nonspecific white matter lesions, OR (b) optic nerve MRI with T2-hyperintense lesion or T1-weighted gadolinium-enhancing lesion extending over >1/2 optic nerve length or involving optic chiasm
2. Acute myelitis: requires associated intramedullary MRI lesion extending over ≩3 contiguous segments (LETM) OR ≩3 contiguous segments of focal spinal cord atrophy in patients with a history compatible with acute myelitis
3. Area postrema syndrome: requires associated dorsal medulla/area postrema lesions
4. Acute brainstem syndrome: requires associated periependymal brainstem lesions

Altered consciousness, decreased attention, impaired memory, and fever are observed in collagen diseases such as SLE, Hashimoto’s encephalopathy, and autoimmune encephalitis [4,5]. These symptoms are similar to those occurring in NMOSD [6,7]. Thus, it is difficult to distinguish NMOSD from other diseases based on symptoms alone. The key to diagnosing NMOSD is the anti-AQP4 antibody. The anti-AQP4 antibody is a specific antibody for NMO/NMOSD and has a sensitivity of 74-83% and a specificity of 100% [8]. Therefore, if the anti-AQP4 antibody is positive, NMOSD can be diagnosed. NMO/NMOSD is also known to be associated with autoimmune diseases, including autoimmune thyroid disease (occurring in 17% of patients), Sjogren’s syndrome (2.0%), SLE (2.0%), ulcerative colitis (2.6%), and idiopathic thrombocytopenic purpura (1.3%) [9]. Thus, if antinuclear, anti-thyroglobulin, or anti-thyroid peroxidase antibodies are positive, the possibility of complications of NMO/NMOSD should be considered.

## Conclusions

We encountered a case of NMOSD with a fever lasting more than three weeks and rapid cognitive decline without positive imaging findings. NMOSD is difficult to diagnose when MRI findings are negative. However, abnormal MRI findings are not essential to the diagnosis of NMOSD, so we cannot rule out NMOSD even if the imaging is normal. To confirm NMOSD, we should check the anti-AQP4 antibody. We should also remember that NMOSD may be associated with various autoimmune diseases that may conceal NMOSD.
